# A safe and effective treatment combination of neoadjuvant therapy and surgical resection for metastatic *TFE3*-rearranged renal cell carcinoma:a case report

**DOI:** 10.3389/fonc.2023.1252282

**Published:** 2023-10-23

**Authors:** Haiyang Yang, Xiang Dong, Xinghe Pan, Wenliang Ma, Jun Pan, Hongqian Guo, Weidong Gan

**Affiliations:** ^1^ Nanjing Drum Tower Hospital Clinical College of Jiangsu University, Nanjing, China; ^2^ Nanjing Drum Tower Hospital, Affiliated Hospital of Medical School, Nanjing University, Nanjing, China; ^3^ Nanjing Drum Tower Hospital Clinical College of Nanjing University of Chinese Medicine, Nanjing, China

**Keywords:** TFE3, translocation, prognosis, renal cell carcinoma, case report

## Abstract

*TFE3*-rearranged renal cell carcinoma (RCC) is a rare subtype of renal tumor that primarily affects young women and is characterized by early metastasis and a poor prognosis. This case study presents a 29-year-old woman diagnosed with *TFE3*-rearranged RCC, who initially presented with painless gross hematuria. Computed Tomography (CT) imaging revealed the presence of a solid mass in the left kidney along with retroperitoneal metastasis. The patient received axitinib, a vascular endothelial growth factor receptor-tyrosine kinase inhibitor (VEGFR-TKI), as first-line neoadjuvant therapy. Subsequent testing confirmed positive expression of programmed death-1 protein L1 (*PDL1*), leading to the addition of tislelizumab, a PD1 inhibitor, to the treatment regimen. After 8 months, the patient’s tumor size and metastases exhibited significant reduction, providing a favorable opportunity for subsequent surgical intervention. The tumor was classified as IV (pT3aN0M1) based on the pathologic stage of the American Joint Committee on Cancer (AJCC, 8th edition, 2017). The patient achieved long-term survival through combined systemic therapy involving surgery and neoadjuvant treatment. At the 30-month follow-up, there was no evidence of tumor recurrence or metastasis.

## Background

In 2004, the World Health Organization (WHO) classified *TFE3*-rearranged renal cell carcinoma (RCC) as a rare and novel subtype of renal cell carcinoma (1). In 2016, along with t (6;11), Xp11.2 translocation was included in the MiT family translocation RCC (2). The most recent WHO classification in 2022 designated it as *TFE3*-rearranged RCC (3). Currently, the primary diagnostic methods for Xp11.2 translocation RCC are *TFE3* fluorescence *in situ* hybridization (FISH) analysis and *TFE3* immunohistochemical (IHC) staining. Genetic sequencing can also be utilized to identify specific molecular subtypes, if available. This subtype predominantly affects individuals under the age of 40, with an overall incidence ranging from 1% to 5%. Although predominantly observed in young patients, the prognosis for *TFE3*-rearranged RCC is worse in the elderly population compared to children (4, 5). Additionally, *TFE3*-rearranged RCC exhibits a higher frequency of lymph node invasion and distant metastasis compared to other renal neoplasms, resulting in an overall poor prognosis even after surgical intervention.

Surgery remains the preferred treatment for renal carcinoma; however, for patients with locally advanced kidney cancer, direct surgery often presents challenges. In recent years, neoadjuvant therapy has gained traction as an effective approach for clear cell renal cell carcinoma (CCRCC). Nevertheless, the efficacy of neoadjuvant therapy in *TFE3*-rearranged RCC, a tumor associated with a poor prognosis, remains uncertain (6, 7). This study presents the case of a patient with metastatic *TFE3*-rearranged RCC who underwent neoadjuvant immune and targeted therapy, resulting in significant tumor shrinkage. Subsequently, the patient underwent surgery and has since maintained a disease-free survival time of 30 months.

## Case presentation

**Figure 1:**
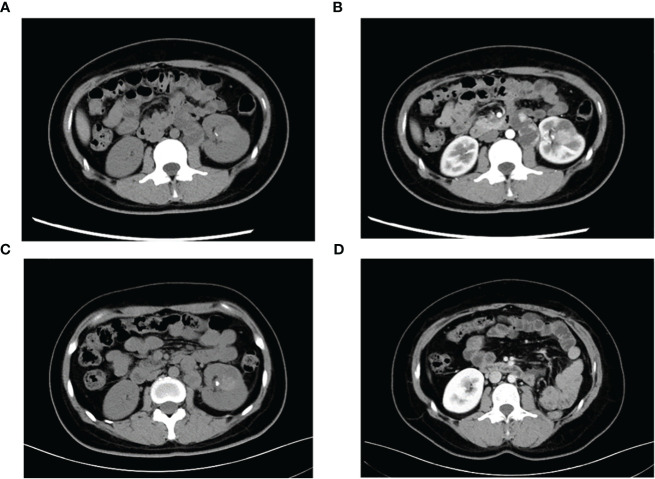
Representative CT images of TFE3-rearranged RCC patients. **(A)** High-density lesions with scattered calcifications (arrows) in the lower pole of the left kidney were observed on plain scan; cystic-solid suspicious metastases (triangles) were observed in the retroperitoneum. **(B)** Moderate enhancement of the left renal lesion and suspicious metastases was observed during the enhanced phase, and the degree of enhancement was lower than that of the adjacent renal cortex. **(C)** Lesions and metastases were significantly reduced after neoadjuvant therapy. **(D)** Postoperative reexamination showed no recurrence of tumor metastasis.

**Figure 2:**
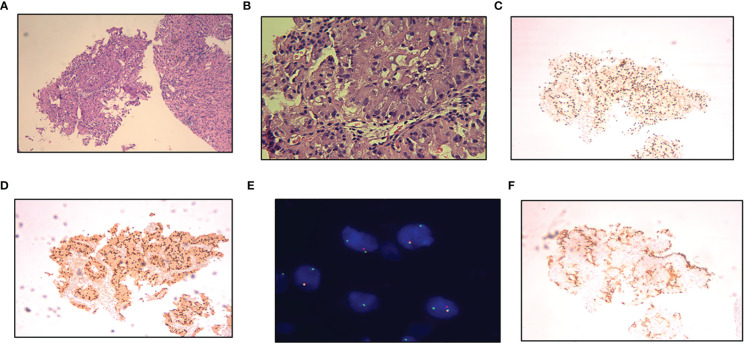
**(A, B)** Pathology of left renal needle biopsy, papillary cells arrangement, marked cellular eosinophilia (H&E, A× 200, B×800). **(C)** TFE3 overexpression (×200). **(D)** Positive for PAX8 (×200). **(E)** Tumor cells have split signals in fluorescence in situ hybridization analysis. One yellow fused signal, one pair of red-green separated signals can be seen. **(F)** Positive for PDL1 (×200).

**Figure 3:**
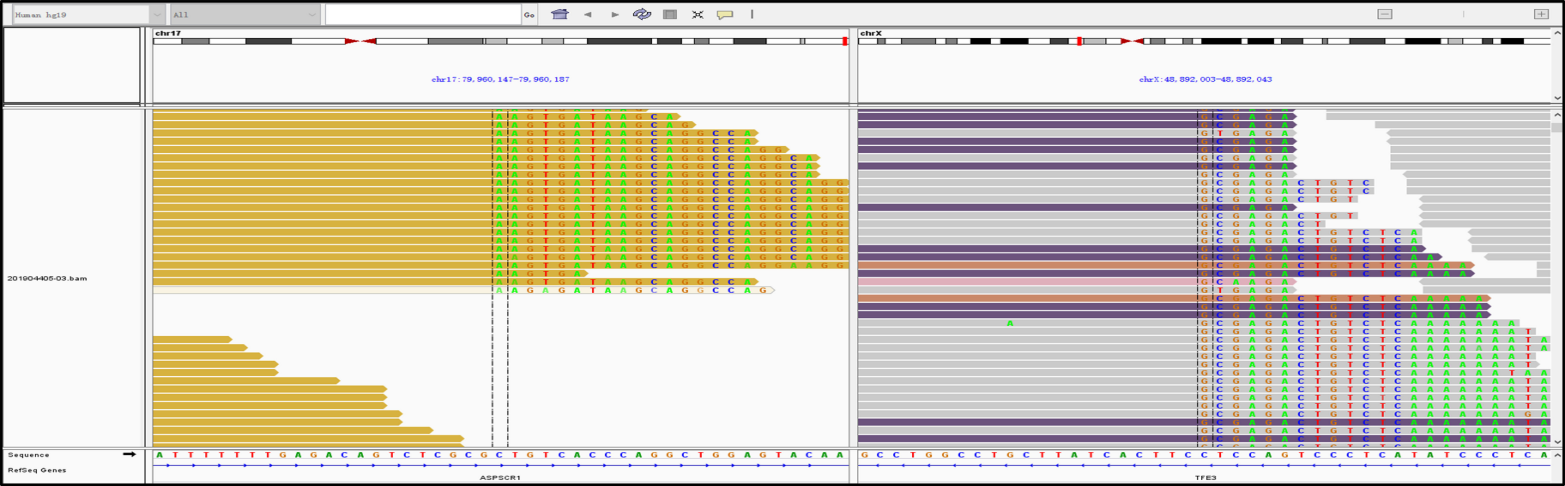
A fusion of TFE3 and ASPSCR1 was detected in the tumor.

A 29-year-old female presented with painless and gross hematuria and no other symptoms or medical history were found. The patient had no prior history of malignancies and the results of urine exfoliative cytology ruled out uroepithelial carcinoma. A computed tomography (CT) scan revealed a solid-cystic tissue density lesion with calcification measuring 3.2 x 2.8 cm at the ventral middle and lower poles of the left kidney (**Figure 1A**). The solid component demonstrated moderate enhancement in the corticomedullary phase (**Figure 1B**). In addition to the primary tumor, a suspected metastasis was observed at the left retroperitoneum with a diameter of approximately 4.6 x 2.4 cm. No metastases present at other sites. Due to the patient’s young age, a specific type of kidney cancer could not be excluded. Therefore, a left renal puncture biopsy was performed, and the pathology morphology was examined using hematoxylin-eosin (HE) staining, which revealed hyaline eosinophilic cytoplasm with papillary structures and psammoma bodies (**Figures 2A, B**). Additionally, strong positive nuclear immunohistochemical staining of *TFE3* was observed (**Figure 2C**). Immunohistochemically, negative results were observed for *CAIX*, *CK7*, and *ALK*, while *CD10* and *PAX8* are positive (**Figure 2D**). *TFE3*-rearranged RCC could not be excluded; thus, a *TFE3* break-apart FISH was performed, which confirmed the diagnosis of *TFE3*-rearranged RCC (**Figure 2E**). To further clarify the molecular type, targeted next-generation sequencing was performed, and the final diagnosis of *ASPSCR1-TFE3* fusion translocation RCC was confirmed (**Figure 3**).

The main indications for neoadjuvant therapy for RCC are: First: High-risk bilateral RCC and isolated RCC. Second: Locally progressive RCC in poor general condition that cannot tolerate surgery for the time being. Third: Patients with high-risk RCC. Fourth: RCC combined with thrombus (8, 9). TFE3-rearranged RCC, as a high-risk tumor, often experiences short-term tumor progression even after patients undergo surgical treatment. Considering that the patient is typically young and the tumor type is quite unique, pure surgical treatment may not achieve satisfactory therapeutic results. In recent years, neoadjuvant therapy has demonstrated promising efficacy in advanced kidney cancer. Therefore, after thorough communication with the patient and their family, it is advisable to consider neoadjuvant therapy as the initial approach rather than immediate surgery. On one hand, preoperative neoadjuvant therapy is expected to reduce the size of the tumor and retroperitoneal metastasis lesions, creating a more favorable time for subsequent surgery. On the other hand, it can provide valuable guidance for postoperative drug treatment choices. The patient underwent neoadjuvant therapy with VEGFR-TKI axitinib (5 mg orally once daily) as first-line treatment to reduce the size of the tumor lesion and metastasis. High *PD-L1* expression was observed in the *PD-L1* immunohistochemistry analysis (**Figure 2F**), which is consistent with previous studies that showed the correlation between *PD-L1* expression levels in tumor tissues and the efficacy of *PD-1* antibody treatment for *TFE3*-rearranged RCC (10, 11). Therefore, we administered the anti-programmed cell death-1 antibody tislelizumab (200 mg intravenously once every 3 weeks) targeted therapy. After 8 months of neoadjuvant therapy, the tumor and metastasis sizes decreased by 16% (3.2 × 2.8 cm vs. 2.7 × 2.4 cm) and 30% (4.6 × 2.4 cm vs. 3.2 × 2.2 cm), respectively, compared to their original sizes (**Figure 1C**). In July and August 2021, the CT scan showed no further decrease in the tumor and metastasis sizes after neoadjuvant therapy. As a result, laparoscopic left radical nephrectomy and retroperitoneal metastasis dissection were performed in August 2021. During the surgery, we did not find any evidence of tumor invasion into the adrenal tissue. Therefore, we did not perform an adrenalectomy.

**Figure 4:**
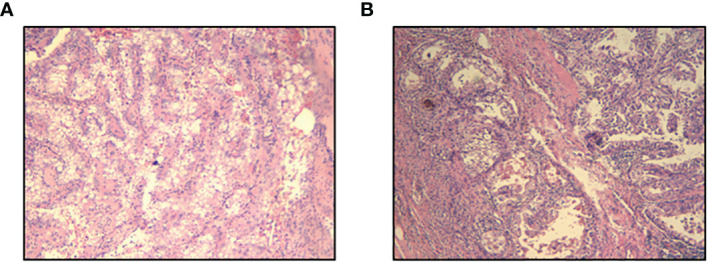
Postoperative pathological view. **(A)** Specimens of masses (×100). **(B)** Retroperitoneal metastases (×200).

**Figure 5:**
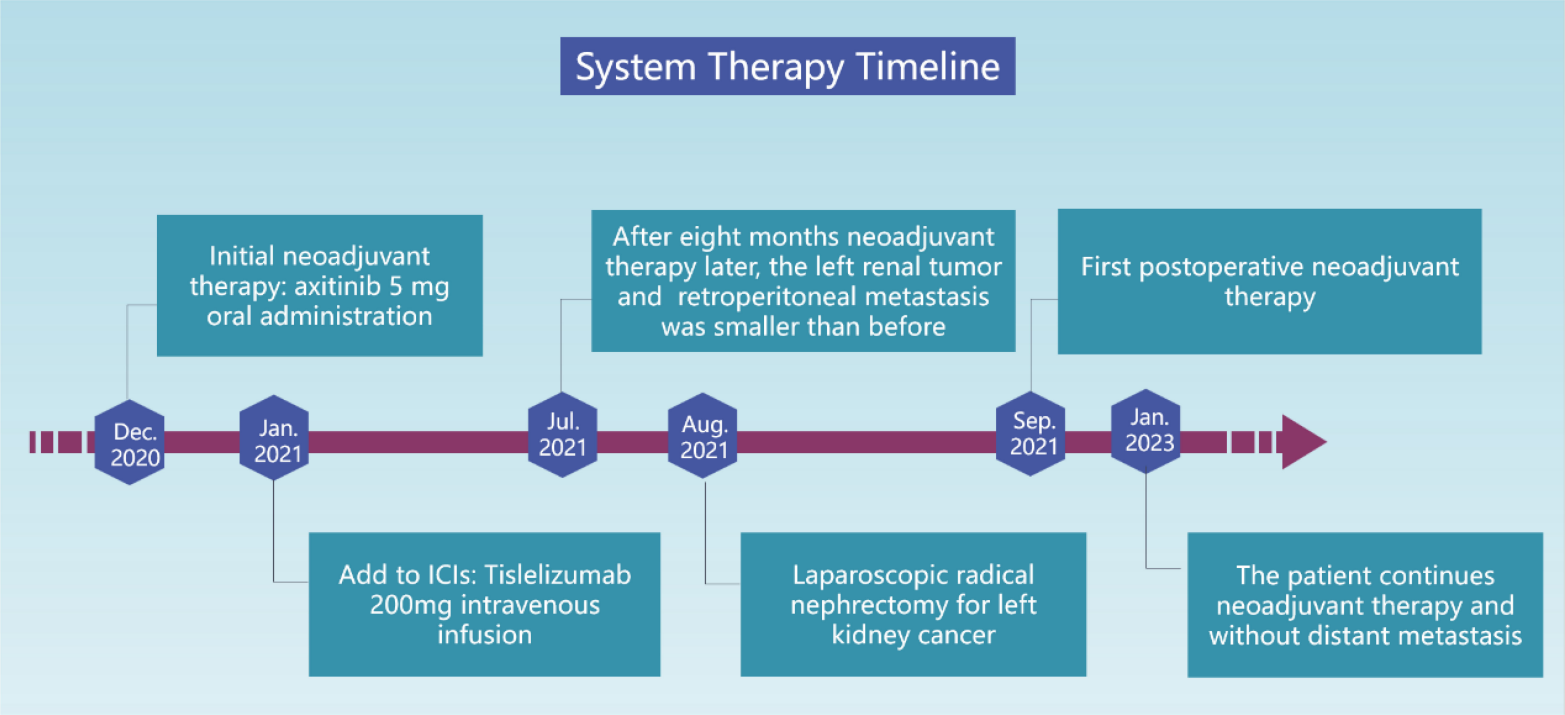
The patient systemic therapy timeline.

The tumor was classified as IV (pT3aN0M1) based on the pathologic stage of the American Joint Committee on Cancer (AJCC, 8th edition, 2017). The postoperative pathology revealed that the tumor was WHO/ISUP grade 4, and it appeared as a distinct papillary structure (**Figure 4A**). The HE staining confirmed that the metastatic foci were composed of tumor tissues (**Figure 4B**). Additionally, the FISH test showed that most tumor nuclei had separated red and green signals, indicating a rearrangement of the *TFE3* gene. The patient suspended tislelizumab therapy due to impaired liver function more than a month after the surgery and continued taking VEGFR-TKI. In September 2021, the patient resumed neoadjuvant therapy with axitinib and tislelizumab. The systemic therapy timeline is presented in **Figure 5**. As of now, the patient has survived for 30 months post-surgery and remains in stable condition without any recurrence of tumor or distant metastases (**Figure 1D**).

## Discussion

Compared to the common CCRCC, this rare *TFE3*-rearranged RCC has stronger invasiveness and poor prognosis. This RCC subtype is defined by different translocations involving chromosome Xp11.2, all of which result in fusion of the *TFE3* gene with other partner genes. So far, an increasing number of fusion types have been discovered. This study reports a female patient diagnosed with *TFE3*-rearranged RCC who was hospitalized for gross hematuria. Previous literature reports have shown that *TFE3*-rearranged RCC predominantly affect children and young adults, with a slightly higher incidence in women (5); *TFE3*-rearranged RCC typically appears as a cystic or solid high-density mass with occasional calcification on CT plain scan. On enhanced scan, the tumor shows moderate enhancement, weaker than the cortex but stronger than the medulla at all stages except the delayed phase (12). The CT imaging of this patient is consistent with previous reports. Based on our previous research, young females with gross hematuria, high-density on non-contrast CT and enhanced enhancement pattern are highly suspicious for Xp11.2 translocation RCC (13). Considering the patient’s clinical and imaging features, the possibility of *TFE3*-rearranged RCC cannot be ruled out, Then, we performed a left renal biopsy on the patient, postoperative morphological staining and *TFE3* immunohistochemistry highly suggested the possibility of *TFE3*-rearranged RCC. Finally, we conducted FISH testing and sequencing to confirm the diagnosis of *TFE3*-rearranged RCC. *TFE3* break-apart FISH analysis is currently considered the gold standard for diagnosing *TFE3*-rearranged RCC, while sequencing can further classify molecular subtypes (14, 15). The case in this study was diagnosed as *TFE3*-rearranged RCC and the existence of *ASPSCR1-TFE3* fusion gene was confirmed by sequencing.

In recent years, neoadjuvant therapies have achieved encouraging therapeutic effects in various types of cancers. Neoadjuvant therapy is considered to be a promising alternative treatment strategy for the Pancreatic Ductal Adenocarcinoma (16). Meanwhile, neoadjuvant therapy has been widely used in breast cancer, and this treatment strategy is mainly used for locally advanced breast cancer without distant metastasis (17). Since the era of cytokines-based immunotherapy, the treatment landscape for metastatic-RCC has profoundly evolved towards targeted agents and novel immunotherapies, greatly improving the prognosis of our patients. Neoadjuvant immunotherapy has a number of advantages over adjuvant immunotherapy, including a potentially stronger immune response to antigens in the tumor, enhanced tumor antigen presentation in the tumor-draining lymph nodes and the lack of a possible immunosuppressed state after surgery (18). In addition, neoadjuvant therapy may increase the chance of nephron-sparing partial nephrectomy in some patients.

Currently, various neoadjuvant treatment options exist for renal cancer, including cytokines, VEGFR-TKI, and immunotherapy. These therapies can facilitate the surgical resection of primary renal tumors (19). Silberstein et al. reported on 12 patients with CCRCC, two of whom had bilateral renal carcinoma. The mean tumor diameter before treatment was 7.1 cm, and after two cycles of sunitinib, the mean tumor size reduction was 1.5 cm (20). In the SURTIME study, patients with advanced renal cell carcinoma who received neoadjuvant sunitinib followed by cytoreductive surgery had a mean overall survival (OS) of 32.4 months, compared to 15.0 months in the direct cytoreductive surgery group (21).This suggests that cytoreductive therapy with preoperative neoadjuvant treatment can potentially extend survival even in patients with metastatic renal cancer. Neoadjuvant therapy for non-CCRCC has been rarely reported, as it is a rare condition and most studies have focused on systemic therapy for advanced metastatic non-CCRCC. Similarly, as a rare subtype of kidney cancer, effective treatment strategies for *TFE3*-rearranged RCC are still under exploration. Although previous studies have reported some therapeutic efficacy of VEGFR-TKIs and mTOR inhibitors for *TFE3*-rearranged RCC, the ultimate treatment outcomes vary to some extent (22–25). In recent years, the use of combination therapy based on immune checkpoint inhibitors (ICIs) has been gradually increasing in clear cell RCC. This also offers new perspectives for the treatment of *TFE3*-rearranged RCC. In a previous study, two metastatic *TFE3*-rearranged RCC patients who received VEGFR-TKI plus ICI as first-line treatment achieved favorable results, with progression-free survival (PFS) exceeding 16.6 months and 25.6 months, respectively (26). However, the application of neoadjuvant therapy for TFE3-rearranged RCC remains limited, Wang et al. reported a case of an adult patient with metastasis (T3aN1M1) resulting from *TFE3*-rearranged RCC. The patient underwent successful surgical resection after targeted therapy, alcohol ablation, and transarterial chemoembolization. No recurrence or metastasis was observed within one year after surgery, and the patient survived for more than 3 years (27).

Axitinib, a second-generation VEGFR-TKI introduced in 2012, offers significant advantages over other TKIs in terms of postoperative complications and tumor shrinkage rate. This is attributed to its high selectivity and shorter half-life, resulting in less intraoperative blood loss (28). In a single-arm phase II clinical study by Karam et al., 24 patients with non-metastatic CCRCC received adjuvant axitinib before surgery. Among them, 11 patients showed partial effectiveness, with a median tumor volume reduction of 28.4%. Thirteen patients exhibited no significant change, and there was no further tumor progression (29). Park et al. reported an objective response rate of 37.5% and a disease control rate of 67.5% for axitinib as adjuvant therapy in patients with metastatic or recurrent non-CCRCC. The median progression-free survival was 10.8 months, demonstrating good efficacy (30); However, there is currently a lack of reports on axitinib as neoadjuvant therapy for non-clear cell carcinoma. Nevertheless, recent large clinical studies have highlighted the significance of immune combined targeted therapy in the treatment of renal carcinoma (31–33). *PD1* inhibitors combined with axitinib have emerged as the recommended first-line treatment for metastatic renal carcinoma (34). A study by Wang et al. showed significant differences in the objective response rate (59.1% vs. 40.7%) and disease control rate (81.8% vs. 40.7%) between axitinib combined with tislelizumab and axitinib alone in advanced renal carcinoma. Moreover, the combined treatment group exhibited higher overall survival (median overall survival 8.9 months vs. 5.8 months) (35). In our case, after diagnosing the patient with *TFE3*-rearranged RCC, considering the patient’s young age and presence of metastases, the prognosis was expected to be poor. Therefore, neoadjuvant therapy to reduce the size of the tumor and metastases was considered prior to surgery. Neoadjuvant axitinib was administered following central evaluation. Subsequently, positive *PDL1* expression was detected through immunohistochemistry, leading to the addition of the immunosuppressant tislelizumab to the treatment regimen. After 8 months of treatment, the tumor and metastases showed reductions of 16% (3.2 × 2.8 cm to 2.7 × 2.4 cm) and 30% (4.6 × 2.4 cm to 3.2 × 2.4 cm), respectively. This response may be attributed to neoadjuvant therapy, indicating some effectiveness in this patient. The patient subsequently underwent radical nephrectomy, which confirmed the diagnosis upon pathological examination. Postoperatively, medication was continued, and during the 30 months follow-up period, no tumor recurrence or metastasis was observed, indicating a favorable long-term prognosis.

## Conclusion

*TFE3*-rearranged RCC is a highly invasive tumor that frequently metastasizes early, resulting in a poor prognosis for patients. For tumors that have progressed to the metastatic stage, neoadjuvant therapy presents a promising treatment approach. In our study, we employed a neoadjuvant treatment regimen consisting of axitinib and tislelizumab. This approach notably reduced the tumor and metastatic lesion volumes before surgery and demonstrated sustained efficacy during long-term follow-up after the surgical procedure.

## Data availability statement

The datasets presented in this study can be found in online repositories. The names of the repository/repositories and accession number(s) can be found in the article/supplementary material.

## Ethics statement

The studies involving humans were approved by Ethics Committee, Nanjing Drum Tower Hospital, Affiliated Hospital of Medical School, Nanjing University. The studies were conducted in accordance with the local legislation and institutional requirements. The participants provided their written informed consent to participate in this study. Written informed consent was obtained from the individual(s) for the publication of any potentially identifiable images or data included in this article.

## Author contributions

HY: project development, manuscript writing and editing, First Author. XD: project development, manuscript writing and editing. WM: project development. XP: pathological data editing. JP: pathological data editing. HG: project development. WG: project development, Corresponding Author. All authors contributed to the article and approved the submitted version.
